# Content analysis of NOC outcomes related to mechanical ventilation in people with COVID-19

**DOI:** 10.1590/1980-220X-REEUSP-2023-0343en

**Published:** 2024-04-05

**Authors:** Erika Silva de Sá, Aline Batista Maurício, Larissa Giardini Bruni, Larissa Gabrielle Dias Vieira, Vinicius Batista Santos, Agueda Maria Ruiz Zimmer Cavalcante, Alba Lucia Bottura Leite de Barros, Viviane Martins da Silva

**Affiliations:** 1 Universidade Federal de Goiás, Goiânia, GO, Brazil.; 2 Universidade Federal de São Paulo, São Paulo, SP, Brazil.; 3 Universidade Federal do Ceará, Fortaleza, CE, Brazil.

**Keywords:** Outcome Assessment, Health Care, COVID-19, Validation Study, Nursing Assessment, Standardized Nursing Terminology, Evaluación de Resultado en la Atención de Salud, COVID-19, Estudio de Validación, Evaluación en Enfermería, Terminología Normalizada de Enfermería, Avaliação de Resultados em Cuidados de Saúde, COVID-19, Estudo de Validação, Avaliação em Enfermagem, Terminologia Padronizada em Enfermagem

## Abstract

**Objective:**

To analyze the evidence of content validity of the Nursing Outcomes “Mechanical Ventilation Response: Adult” and “Mechanical Ventilation Weaning Response: Adult”, for patients with severe COVID-19.

**Method:**

Methodological study developed in two stages: literature review to construct the definitions of the indicators and analysis of the evidence of content validity of the nursing outcomes by a focus group.

**Results:**

All the conceptual and operational definitions developed for the 56 indicators were considered clear and precise. However, 17 indicators were excluded because they were deemed not to be relevant. The definitions of the magnitudes for 17 indicators of the Nursing Outcome “Mechanical Ventilation Response: Adult” and 22 indicators “Mechanical Ventilation Weaning Response: Adult” were thus constructed.

**Conclusion:**

The development of definitions and validation by experts makes the use of these outcomes and their indicators more understandable and precise, favoring their use in clinical practice and providing greater detail in assessment and recording.

## RESUMO

**Objetivo:**

Analisar as evidências de validade de conteúdo dos Resultados de Enfermagem “Resposta à ventilação mecânica: adulto” e “Resposta ao Desmame da Ventilação Mecânica: adulto”, para pacientes adultos com COVID-19 grave.

**Método:**

Estudo metodológico desenvolvido em duas etapas: revisão da literatura para construção das definições dos indicadores e análise das evidências de validade de conteúdo dos resultados de enfermagem por um grupo focal.

**Resultados:**

Todas as definições conceituais e operacionais elaboradas para os 56 indicadores foram consideradas claras e precisas. Entretanto, 17 indicadores foram excluídos por serem julgados pouco relevantes. Desse modo foram construídas as definições das magnitudes para 17 indicadores do Resultados de Enfermagem “Resposta a ventilação mecânica: adulto” e de 22 indicadores “Resposta ao desmame da ventilação mecânica: adulto”.

**Conclusão:**

A elaboração das definições e a validação por especialistas tornam o uso desses Resultados e seus indicadores mais compreensível e preciso, favorecendo o uso na prática clínica, proporcionando maior detalhamento da avaliação e dos registros.

## RESUMEN

**Objetivo:**

Analizar la evidencia de validez de contenido de los resultados de enfermería “Respuesta a la ventilación mecánica: Adulto” y “Respuesta al destete de la ventilación mecánica: Adulto”, para pacientes adultos con COVID-19 grave.

**Método:**

Estudio metodológico desarrollado en dos etapas: revisión bibliográfica para construir las definiciones de los indicadores y análisis de la evidencia de validez de contenido de los resultados de enfermería mediante un grupo focal.

**Resultados:**

Todas las definiciones conceptuales y operativas elaboradas para los 56 indicadores se consideraron claras y precisas. Sin embargo, se excluyeron 17 indicadores porque se consideró que no eran pertinentes. Se construyeron así las definiciones de las magnitudes para 17 indicadores del resultado de enfermería “Respuesta a la ventilación mecánica: adulto” y 22 indicadores “Respuesta al destete de la ventilación mecánica: adulto”.

**Conclusiones:**

La elaboración de definiciones y validación por expertos hace más comprensible y preciso el uso de estos resultados y sus indicadores, favoreciendo su uso en la práctica clínica y aportando mayor detalle en la valoración y registro.

## INTRODUCTION

The COVID-19 (Coronavirus Disease 2019) pandemic has brought a healthcare reality in which decisions must be made quickly^(1)^. COVID-19 is a disease caused by the SARS-CoV-2 virus with clinical manifestations that involve all systems, especially the respiratory system^(2)^.

Patients who develop the severe or critical form of the disease may require hospitalization in an Intensive Care Unit (ICU) and invasive ventilatory support^(2)^. Nursing care for these patients is complex and requires clinical reasoning (CR). Therefore, it is essential to carry out CR anchored in the Nursing Process (NP) using standardized language^(3)^.

In their clinical practice, during the planning stage, nurses must establish the expected results for the patient’s evolution and evaluate them as the interventions are carried out^(4)^.This continuous evaluation can be carried out using the Nursing Outcomes Classification (NOC), which makes it possible to analyze the patient’s clinical evolution and determine changes in the interventions implemented, to achieve the expected result^(4)^.

The NOC outcomes describe a state, behavior or perception of the individual, family or community, using a 5-point Likert scale, and can evaluate the individual’s responses to nursing interventions, measuring them along a continuum^(4)^.

However, the NOC does not provide conceptual definitions (CD) and operational definitions (OD) for its indicators^(5)^, which makes the evaluation subjective. Therefore, for the Nursing Outcomes Classification (NOC) to be useful in clinical practice, content validation studies are needed to improve their structure and make the outcome indicators clearer and more understandable^(4)^.

Faced with the need to assess patients with severe COVID-19 hospitalized in the ICU, under invasive mechanical ventilation and subsequent ventilatory weaning, no studies of evidence of content validity of the NOC outcomes “Mechanical Ventilation Response: adult” and “Mechanical Ventilation Weaning Response: adult” were identified in the literature. It is believed that, given the similar clinical manifestations caused by SARS-CoV-2 and acute respiratory distress syndrome (ARDS), the validation of these Outcomes can optimize care, allow more precise and targeted approaches to mitigate respiratory complications, improving clinical outcomes for this population. Building definitions for the NOC outcome indicators and assessing the clarity and precision of their content can guide decision-making by nurses and guide actions in the face of serious clinical conditions, aiming for quality and safety for professionals and patients, as well as making it possible to measure the clinical evolution of these patients to achieve better health outcomes.

The objectives of this study were to analyze the evidence of content validity of the Nursing Outcomes “Mechanical Ventilation Response: adult (0411)” and “Mechanical Ventilation Weaning Response: adult (0412)”.

## METHOD

### Study Design

This methodological study was carried out in two stages: the first was a literature review to develop CD and OD for each indicator, as well as OD for each level of magnitude of the Nursing Outcome scales under study. Subsequently, the indicators and their respective definitions were submitted for evaluation by judges through a focus group (FG) for content analysis.

### Population, Selection Criteria, and Data Collection

In the first stage, a literature review was carried out following the recommendations of the PRISMA Extension for Scoping Reviews (PRISMA-ScR): Checklist and Explanation^(5)^, and the databases Latin American and Caribbean Literature in Health Sciences (LILACS); MEDLINE via PubMed; Cumulative Index to Nursing and Allied Health Literature (CINAHL); Brazilian Nursing Database (BDENF); SCOPUS; Web of Science (WOS) and Excerpta Medica Database (EMBASE) were consulted to identify relevant and useful documents for the process of drawing up the conceptual and operational definitions. To identify the studies, we typed in terms referring to each of the indicators and connected them to the Boolean operators AND/OR according to the specificity of each database (DeCS, MeSH terms, CINAHL titles), considering the indicators of both Nursing Outcomes NOC. It was also necessary to use textbooks, Mechanical Ventilation guidelines^(6)^, and the official websites of specialized societies. The search for databases to prepare the definitions took place between July and December 2020, without the publication of the protocol, although the information from this search could be made available by the corresponding author.

The inclusion criteria were: primary studies, complete and available online, in the following languages: Portuguese, English, or Spanish, with no time limit, and which dealt with Nursing Outcomes indicators. Letters, editorials, and studies which, although they addressed the indicator, did not provide definitions or characteristics about them were excluded.

For the second stage of the study, four judges were selected for convenience, who were members of the Nursing Process Research Network (RePPE), with a degree in nursing, professors, and researchers with research into nursing terminology. All the judges were classified as senior experts, achieving a score of more than 20 points, according to the following criteria and scores: clinical experience of at least four years in the specific area (04 points), teaching experience of at least one year in the specific area and teaching nursing classifications (01 point), research experience with published articles on nursing classifications in reference journals (01 point), participation of at least two years in a research group in the specific area (01 point), a doctorate in nursing in the specific area (02 points), master’s degree in nursing in the specific area (01 point) and residency in nursing in the specific area (01 point)^(7)^. This strategy aimed above all to ensure a high degree of technical and scientific qualification in the FG discussions, as well as a thorough understanding of the indicators for both outcomes.

### Data Analysis and Treatment

Based on the literature review, CDs and ODs were drawn up for all the indicators, as well as for each level of magnitude of the Nursing Outcomes scale under study. In the second stage, the judges assessed the relevance of the indicators, as well as the clarity and precision of the CDs and ODs and the definitions of the magnitudes. Relevance corresponds to the item’s ability to be consistent with the defined attribute and with other expressions relating to the same attribute. Clarity is the ability of the item to be intelligible, with short sentences, simple expressions (presenting a single idea), and unambiguous. Precision is the ability of the item to have a defined position and be distinct from other items that refer to the same concept^(8)^.

The judges assessed the relevance of the indicator without considering a specific population. Likewise, the CDs were constructed from this perspective. On the other hand, the ODs and magnitudes in this study were constructed and assessed regarding the population of critically ill adult patients with severe COVID-19.

The FG technique was used to assess the criteria of the relevance of the indicators, the clarity and precision of the DCs, and the definitions of the magnitudes^(9)^. The instrument was made available to the judges via email, containing the indicators, CDs, and ODs, approximately 15 days before each meeting.

Five meetings were held via the Google Meet platform, each lasting approximately 3 hours. The relevance of the indicators and the clarity and precision of the definitions were assessed based on the discussions, with the definitions being reworked, when necessary, to better represent the phenomenon being assessed. The discussions were led by a moderator with expertise in standardized language research, accompanied by two other observers who helped lead the sessions.

During the meetings, the title of the indicator, the CD, OD, and magnitudes were read out. At the first meeting, the indicators were assessed for their relevance (relevant, not very relevant, and irrelevant). In the following meetings, the CD and OD were assessed for clarity (clear, unclear, and unclear) and precision (precise, imprecise, and imprecise). Each definition was discussed at length; when it was not considered precise or clear by at least one judge, it was reformulated by the researchers after the meetings and resubmitted to the judges’ evaluation at the next meeting until an absolute consensus was reached.

### Ethical Aspects

The project was cleared by the Human Research Ethics Committee of the Federal University of São Paulo, under protocol number 4.251.184, in 2020. All participants signed an informed consent form to take part in the study complying with all the guidelines set out in Resolution 466/2012.

## RESULTS

The literature review made it possible to compose the CD and OD for the clinical indicators, as well as for the magnitude of the scale. All the indicators were submitted for evaluation by the judges to check their relevance, clarity, and precision. The judges made suggestions for various definitions to improve them and make them clearer for subsequent clinical evaluation. All the indicators were considered relevant. Among the indicators evaluated for the “Mechanical Ventilation Response: adult” and “Mechanical Ventilation Weaning Response: adult”, 17 indicators and 22 indicators, respectively, had their conceptual and operational definitions considered clear and precise, as shown in Chart 1.

**Chart 1:** Indicators of “Mechanical Ventilation Response: adult” and “Mechanical Ventilation Weaning Response: adult” whose definitions were considered clear and precise in the judges’ assessment – Goiânia, GO, Brazil, 2023.

“Mechanical Ventilation Response: adult”	“Mechanical Ventilation Weaning Response: adult”
Respiratory rate	Spontaneous respiratory rate
Respiratory rhythm	Spontaneous respiratory rhythm
Depth of inspiration	Spontaneous respiratory depth
Respiratory secretions	Apical heart rate
Fraction of inspiratory oxygen (FiO_2_) that meets oxygen demand	Partial pressure of oxygen in arterial blood (PaO_2_)
Partial pressure of oxygen in arterial blood (PaO_2_)	Partial pressure of carbon dioxide in arterial blood (PaCO_2_)
Partial pressure of carbon dioxide in arterial blood (PaCO_2_)	Arterial pH
Arterial pH	Oxygen saturation
Oxygen saturation	Tidal volume
Peripheral tissue perfusion	Minute ventilation < 10 L/minute
Tidal volume	Positive end-expiratory pressure
Chest X-ray	Chest X-ray findings
Difficulty breathing with ventilator	Respiratory secretions
Adventitious breath sounds	Anxiety
Atelectasis	Fear
Impaired skin integrity at tracheostomy site	Impaired cough reflex
Pulmonary infection	Adventitious breath sounds
	Atelectasis
	Restlessness
	Discomfort
	Difficulty communicating needs
	Difficulty breathing on own

Source: Authors (2023).

The title of 7 indicators was changed as suggested by the judges to describe, clarify, and evaluate the phenomenon, as shown in Chart 2.

**Chart 2:** Indicators whose titles were changed as suggested by the judges – Goiânia, GO, Brazil, 2023.

Current title of the indicator according to NOC	Indicator title after judges’ evaluation
Depth of inspiration	Depth of breathing
Chest X-ray findings	Imaging findings
Impaired skin integrity at the tracheostomy site	Impaired skin integrity related to respiratory devices
Difficulty breathing on own	Difficulty maintaining spontaneous ventilation
Discomfort	Pain
Minute ventilation < 10 L/minute	Minute volume
Agitation	Restlessness

Source: Authors (2023).

A three-point scale was also recommended for the indicators: “respiratory rhythm”, “depth of breathing”, “spontaneous respiratory rhythm”, “depth of spontaneous breathing”, and “difficulty communicating needs”; while a four-point scale was recommended for the indicator: “respiratory secretions”. This suggestion was made to assess these indicators more appropriately and precisely, since the grading of an item should be adjusted to its characteristic, representing the alterations commonly described and measured in clinical practice.

For 14 indicators, despite their definitions being considered clear and precise, there was a recommendation to exclude the assessment of patients with severe COVID-19, using mechanical ventilation and hospitalized in the ICU due to the clinical characteristics of the patient, the unavailability of accurate and validated methods to assess the indicator and/or the need for complex and/or expensive technological equipment and devices, unavailable for use in this study. These indicators and their respective justifications are shown in Chart 3.

**Chart 3:** Indicators not recommended and the reasons for not recommending them – Goiânia, GO, Brazil, 2023.

NOC	Indicator	Justification
“Mechanical Ventilation Response: adult”	Anxiety	Impossibility of assessing the indicator due to high doses of sedo-analgesia and neuromuscular blockers.
	Restlessness	
	Difficulty communicating needs	
	Ventilation perfusion balance	Impossibility of assessing this indicator without the aid of a Swan-Ganz catheter, although its use is not common in clinical practice.
	Inspiratory capacity	Indicators cannot be assessed without the respective equipment: ventilometer, spirometer and capnograph.
	Pulmonary function tests	
	End tidal carbon dioxide	
	Hypoxia	The occurrence of silent hypoxia is common in patients with COVID-19 and oxygenation is assessed by oximetry in another indicator.
“Mechanical Ventilation Weaning Response: adult”	Ventilation Perfusion balance	It is impossible to assess this indicator without the aid of a Swan-Ganz catheter, although its use is not common in clinical practice.
	Impaired drive to breath	Impossibility of assessing changes in the autonomic nervous system in patients with COVID-19, in MV and under analgesia.
	Impaired gag reflex	Impossibility of assessing the swallowing reflex since the patients, even when weaned from MV, would still be connected to the ventilator.
Common indicators for both outcomes	Vital capacity	Unable to assess this indicator without a ventilometer.
		Unavailability of a tool to recognize this indicator in mechanically ventilated patients
	Asymmetrical chest wall expansion	

Source: Authors (2023).

The Chart 4 below shows an example of the CD, OD, and magnitude of a common clinical indicator for the two Nursing Classification Outcomes evaluated in this study.

**Chart 4:** Indicator with CD, OD, and magnitude – Goiânia, GO, Brazil, 2023

Indicator	Magnitude				
pH arterial	1 Severe deviation	2 Substantial deviation	3 Moderate deviation	4 Mild deviation	5 No deviation
Conceptual definition: A term that refers to the concentration of hydrogen ion (H+) dissolved in arterial blood. Operational definition: This indicator will be obtained by consulting the values in the most recent blood gas analysis (last 24 hours).	< 6,8 or > 8,0	7,07–6,9 or 7,84–7,99	7,08–7,20 or 7,70–7,83	7,21–7,34 or 7,46–7,69	7,35–7,45

Source: Authors (2023).

## DISCUSSION

The CD and OD were constructed for all 56 indicators of the “Mechanical Ventilation Response: adult” and “Mechanical Ventilation Weaning Response: Adult”. Of these, 39 indicators had clear and precise definitions for patients diagnosed with COVID-19 and on invasive mechanical ventilation.

Of the 56 indicators that were approved and recommended, nine are common to both outcomes “respiratory secretions”, “partial pressure of oxygen in arterial blood (PaO_2_)”, “partial pressure of carbon dioxide in arterial blood (PaCO_2_)”, “arterial pH”, “oxygen saturation”, “tidal volume”, “Chest x-ray findings (imaging findings)”, “adventitious breath sounds” and “atelectasis”^(4)^.

The validated “Mechanical Ventilation Response: adult” indicators were: “respiratory rate”, “respiratory rhythm”, “depth of inspiration”, “fraction of inspired oxygen (FiO_2_) meets oxygen demand”, “peripheral tissue perfusion”, “difficulty breathing with ventilator”, “impaired skin integrity at related to respiratory devices” and “pulmonary infection”. While for the “Mechanical Ventilation Weaning Response: adult” - “spontaneous respiratory rate”, “spontaneous respiratory rhythm”, “spontaneous respiratory depth”, “positive end-expiratory pressure”, “minute ventilation <10 L/min (minute volume)”, “apical heart rate”, “impaired cough reflex”, “anxiety”, “fear”, “agitation (restlessness)”, “discomfort (pain)”, “difficulty communicating needs” and “difficulty breathing on own”.

Among the indicators assessed during the respiratory physical examination are respiratory rate, respiratory rhythm, depth of inspiration, respiratory secretions, and adventitious breath sounds. The first three are assessments made during anamnesis and monitoring of vital signs and this information is useful in subsequent decisions, as it determines respiratory stress, fatigue of accessory muscles, and impaired gas exchange. In patients using mechanical ventilation with a tracheal tube, the primary mechanisms for eliminating respiratory secretions are impaired. In addition, respiratory secretion samples from patients on mechanical ventilation with COVID-19 are typically colored, opaque, viscous, tenacious, with a significantly high percentage of solids. The concentration of proteins, DNA, and hyaluronan of the COVID-19 virus in respiratory secretion samples is higher when compared to healthy people. These characteristics impair the mucociliary clearance of secretions, resulting in an accumulation of fluids in the lungs, hindering their elimination and impairing gas exchange^(10)^.

The auscultation of respiratory sounds is a valuable technique, leading to the identification of sounds with a different intensity and frequency than normal^(11)^. An abnormality in the auscultated sound usually indicates inflammation, infection, obstruction or fluid in the lungs^(11)^. In patients diagnosed with COVID-19, the auscultated adventitious sounds can be of different types such as wheezing, rales, snoring, pleural friction and crackles. These overlap with vesicular murmurs^(12)^.

Indicators directly related to ventilation should be monitored to assess the patient’s dependence on the device. The different objectives in the use of mechanical ventilation are represented by the parameters established. Parametric recommendations should be followed by professionals such as: setting the ventilator to a low tidal volume (4-8 mL/kg); higher PEEP (positive end-expiratory pressure) should be favored (>10 cm/H_2_O) and titrated according to FiO_2_ to obtain an appropriate SpO_2_^(13)^.

Tidal volume, minute ventilation, and positive end-expiratory pressure (PEEP) are observed in the mechanical ventilator parameters. Mechanical ventilation can be used in different clinical situations in the face of respiratory failure and in the case of COVID-19, severe hypoxemia is the main reason. A scoping review found that the parameters used in the mechanical ventilator for COVID-19 patients were similar and followed evidence-based recommendations for lung-protective ventilation^(14)^.

Tidal volume (volume of air in inspiration/expiration during each breath) and respiratory rate establish the minute ventilation. High volumes lead to the risk of pulmonary hyperinflation while a low volume can trigger atelectasis. A high rate can cause hyperventilation and respiratory alkalosis. Conversely, a low rate poses the risk of inadequate minute ventilation and respiratory acidosis. A low tidal volume - calculated from body weight (6 to 8 mL/kg of ideal body weight) - is recommended for patients with acute respiratory distress syndrome (ARDS), and this criterion is also used in COVID-19 patients. The minute ventilation must be appropriate for the patient, but it is considered that volumes greater than 10L in adults occur with great respiratory muscle work and are criteria used to contraindicate ventilatory weaning^(15)^.

Another parameter refers to PEEP and its maintenance aims to minimize or prevent the cyclical collapse of the lungs at the end of inspiration. According to the mechanical ventilation guideline^(6)^, there is no consensus on the values considered ideal, so it should be equal to or less than 8 cm of H_2_O, as this is close to physiological PEEP.

Indicators such as pH, PaO_2_, PaCO_2,_ and SaO_2_ can be assessed using a blood gas test. This helps to assess and adjust the patient’s ventilatory status (PaCO_2_), acid-base status (pH) and oxygenation status (PaO_2_ and oxyhemoglobin saturation), as well as oxygen transport capacity (PaO_2_, oxyhemoglobin saturation, total hemoglobin, and dyshemoglobin) and intrapulmonary shunt. It also helps to determine the degree of hypoxemia by classifying the severity of acute respiratory distress syndrome by calculating the PaO_2_/FiO_2_ ratio, and to monitor the severity and progression of cardiopulmonary diseases, as in the case of COVID-19^(16)^.

The presence of hypoxemia and changes in pH during admission showed an important relationship with the severity of the disease^(17)^. In addition, the presence of dyspnea on admission, PaO_2_ lower than 60 mmHg, and SaO_2_ values lower than 90% influenced the length of hospitalization of people with COVID-19^(18)^.

Oxygenation is most assessed by calculating the ratio of PaO_2_ and the fraction of inspired oxygen (PaO_2_/FiO_2_ or P/F ratio). However, to quantify ineffective gas exchange, this ratio is not accurate, since PaO_2_ varies depending on the oxygen supplied^(19)^. On the other hand, SaO_2_/FiO_2_ is a useful index in the assessment, enabling decision-making without the need for invasive measures, showing better values in predicting mortality from COVID-19^(20)^, and aiding decision-making. In the absence of gasometry, SaO_2_ should be used as a tool and is considered the fifth vital sign^(20)^.

Regarding the outcome “Response to Weaning from Mechanical Ventilation: adult”, the indicators assessed should reveal clinical improvement in the respiratory pattern and level of consciousness and avoid reintubation. One of the main reasons for reintubation is patients’ inability to expectorate secretions^(21)^, which can be influenced by muscle weakness acquired in the ICU (ICU-AW). ICU-AW is a multifactorial clinical condition commonly associated with underlying pathologies, polyneuropathy, myopathy and/or muscle atrophy characterized as generalized muscle weakness. ICU-AW has been found in up to 45.5% of patients with severe COVID-19, and the risk factors for its development are pre-existing diseases, sepsis, shock, multiple organ failure, prolonged mechanical ventilation, immobilization, neuromuscular blockade, corticosteroid use and hyperglycemia^(2)^.

Another indicator that can be influenced by ICU-AW is the impaired cough reflex, which is a predictor of extubation failure. Cough strength can be measured by peak cough flow and the white card test (WCT) by a scale of 0 to 4/5 which measures the progression of the cough, from complete absence to maximum intensity, both methods assessing the affectivity of the cough. A systematic review assessed extubation failure using these two tests. The peak flow cough test showed extubation failure rates of 36.2% and 6.3%, and the WCT scale showed extubation failure rates of 37.1% and 11.3%, respectively, in patients with weak and strong coughs. Both tests showed moderate diagnostic power to predict extubation failure^(22)^.

Evaluation of the “impaired cough reflex” indicator is an important tool in clinical practice, as it demonstrates the individual’s capacity for autonomous control of secretions and is an effective indicator of weaning success and clinical improvement.

Chest CT scans and chest X-rays are other devices used to assess the degree of lung commitment and monitor the clinical course of COVID-19 patients. The extent of lung commitment is a predictive factor of greater severity and mortality. The most common tomographic findings in severe cases consist of ground-glass opacity (92.5%), consolidation (79.2%), parenchymal bands (50%), septal thickening (43.5%), mosaic paving pattern (23.9%) and inverted halo sign (3.5%)^(23)^. Although less sensitive than chest CT, X-rays are useful in assessing pneumonia, pleural effusion or pulmonary edema, and the most common abnormalities are consolidation (28%) and ground-glass opacities (29%). The distribution is most frequently bilateral (43%), peripheral (51%) and in the basal zone (56%)^(23)^.

It is also possible to identify atelectasis on imaging. A retrospective study involving 237 patients showed that up to 24% had atelectasis. Compared to patients with no, small or large atelectasis, the group with greater involvement of this pulmonary condition had a worse SaO_2_/FiO_2_ ratio, the need for mechanical ventilation, a higher rate of ICU admission, length of stay, and mortality^(24)^. Continuous evaluation of this indicator using imaging tests helps to identify and institute early treatment to reverse atelectasis and prevent its progression^(24)^.

As for the “impaired skin integrity related to respiratory devices” indicator, which replaced the “impaired skin integrity at tracheostomy site” indicator, this seems to be more representative of assessing people on mechanical ventilation. Prolonged orotracheal intubation, as well as safety devices for securing the tube, prone positioning, the use of sedatives and vasopressors, and the pathophysiology related to COVID-19, increase immobility in bed, corroborating the risk of developing facial pressure injury^(25)^. In addition, severe SARS CoV-2 infection can induce endothelial dysfunction resulting in cytokine storm, hypercoagulation, and hypoxia causing microthrombosis, increased soft tissue fragility, and reduced tissue perfusion^(25)^.

The prevalence of pressure injuries in patients with COVID-19 who required intensive care was three times higher than that observed in patients without COVID-19^(26)^. These facial injuries associated with respiratory devices have a significant impact on increasing the length of hospital stay, as well as interfering with ventilatory therapy since it reduces tolerance to ventilatory support, increases resistance, patient-ventilator asynchronies, increases the risk of system leakage and decreases therapeutic efficacy^(25)^. Therefore, assessing the risk of developing injuries related to respiratory devices, rigorously monitoring skin integrity as a preventative measure, maintaining proper decubitus, and protecting the most vulnerable areas are necessary interventions to achieve the best nursing care.

Patients in the ICU can experience different intensities of pain during rest and procedures. Severe pain can induce various stress responses such as agitation, sleep disturbances, delirium, tachycardia, increased myocardial oxygen consumption, hypercoagulation, respiratory impairment, immunosuppression, and increased catabolism, leading to tissue perfusion disorders^(27)^.

A cohort of patients admitted to the ICU for ARDS due to COVID-19 recorded a rate of 69.3% of patients in an agitation state^(28)^. Restlessness (Agitation) can lead to difficulties in mechanical ventilation, and cause hypoxia due to increased oxygen consumption, barotrauma, hypotension, and accidental removal of health support devices^(27)^. In addition, critically ill patients in the ICU on mechanical ventilation often have difficulties communicating their needs due to intubation, tracheostomy, ICU-AW due to critical illness, level of sedation, delirium, and others^(29)^. In this context, patients have reported physical and emotional responses such as hopelessness, anxiety, high levels of frustration, and stress as a result of impaired communication and illness^(29)^. Ineffective communication between mechanically ventilated patients and healthcare professionals can interfere with planning and expected outcomes.

The indicators pain (discomfort), anxiety, restlessness, and fear can trigger physiological responses, such as changes in heart rate, respiratory rate and SaO_2_, leading to the need for a higher oxygenation rate, which can delay the weaning process and, consequently, lengthen treatment^(27)^.

The nurse’s assessment uses different indicators that reflect information related to health/disease conditions. Identifying patient problems is important for planning care and monitoring clinical changes, contributing to effective management, patient safety and reducing adverse events.

A limitation was the restriction of languages, including solely studies that were available online and in Portuguese, English or Spanish. Also, as some indicators involved physiological aspects, the gray literature was used extensively. As COVID-19 is a relatively new disease, the conceptual and operational definitions of the clinical indicators were constructed based on studies that addressed the pathophysiology of ARDS, with the main aim of elucidating and assessing its magnitude. However, we emphasize that both NOC results must be clinically validated to demonstrate their applicability.

## CONCLUSION

This study constructed and consensually validated the CD and OD for the “Mechanical Ventilation Response: adult” and “Mechanical Ventilation Weaning Response: adult”. Based on the results of this study, it is possible to develop care protocols for patients on mechanical ventilation, including guidelines for monitoring, measuring, and assessing severity, and directing nursing interventions, even for those professionals with less experience. Therefore, these results can also be used to train teams, enabling them to offer better health practices. Monitoring the patient using these validated indicators is essential to identify improvement, stagnation, or worsening of the clinical condition and to intervene promptly. Clinical validation studies verifying the usefulness of these indicators in practice should be carried out, contributing to the nursing evidence base.
